# Accelerated Aging Behavior in Alkaline Environments of GFRP Reinforcing Bars and Their Bond with Concrete

**DOI:** 10.3390/ma14195700

**Published:** 2021-09-30

**Authors:** Arnaud Rolland, Karim Benzarti, Marc Quiertant, Sylvain Chataigner

**Affiliations:** 1 Département Mobilités et Infrastructures, Cerema Ouest, F-44262 Nantes, France; 2 Lab Navier, University Gustave Eiffel, Ecole Nationale des Ponts et Chaussées (ENPC), Centre National de la Recherche Scientifique (CNRS), F-77447 Marne la Vallée, France; karim.benzarti@univ-eiffel.fr; 3 Matériaux et Structures (MAST) Department, Expérimentation et Modélisation pour le Génie Civil et Urbain (EMGCU), University Gustave Eiffel—Institut Français des Sciences et Technologies des Transports, de l’Aménagement et des Réseaux (IFSTTAR), F-77447 Marne-la-Vallée, France; marc.quiertant@univ-eiffel.fr; 4 Matériaux et Structures (MAST) Department, Structures Métalliques et Câbles (SMC), University Gustave Eiffel—Institut Français des Sciences et Technologies des Transports, de l’Aménagement et des Réseaux (IFSTTAR), F-44344 Bouguenais, France; sylvain.chataigner@univ-eiffel.fr

**Keywords:** glass fiber-reinforced polymer (GFRP) rebars, durability, accelerated aging, tensile strength, interlaminar shear strength (ILSS), bond behavior

## Abstract

This study investigates the durability of glass fiber-reinforced polymer (GFRP) reinforcing bars (rebars) and their bond in concrete. Accelerated aging tests were first conducted on bare rebars that were either subjected to direct immersion in an alkaline solution or previously embedded in concrete before immersion in the solution (indirect immersion). Accelerated aging was conducted at different temperatures of the solution (20 °C, 40 °C and 60 °C) and for various periods up to 240 days. Residual tensile properties were determined for rebars subjected to direct immersion and served as input data of a predictive Arrhenius model. A large decrease in the residual tensile strength assigned to the alkali-attack of glass fibers was extrapolated in the long term, suggesting that direct immersion is very severe compared to actual service conditions. Short-beam tests were also performed on rebars conditioned under direct/indirect immersion conditions, but did not reveal any significant evolution of the interlaminar shear strength (ILSS). In a second part, bond tests were performed on pull-out specimens after immersion in the alkaline solution at different temperatures, in order to assess possible changes in the concrete/GFRP bond properties over aging. Results showed antagonistic effects, with an initial increase in bond strength assigned to a confinement effect of the rebar resulting from changes in the concrete properties over aging, followed by a decreasing trend possibly resulting from interfacial degradation. Complementary characterizations by scanning electron microscopy (SEM), differential scanning calorimetry (DSC) and Fourier transform infrared (FTIR) spectroscopy were also carried out to evaluate the effects of aging on the physical/microstructural properties of GFRPs.

## 1. Introduction

The use of glass fiber-reinforced polymer (GFRP) reinforcing bars (rebars) as a replacement for traditional steel reinforcement is an attractive solution to prevent the corrosion of reinforced concrete (RC) structures, which is the main pathology encountered on civil infrastructures worldwide. Although GFRP rebars have been increasingly used since the 1990s, there is still a lack of knowledge regarding their long-term durability in the alkaline concrete environment.

Humidity and hydroxyl ions coming from the concrete pore medium rich in alkalis, are indeed likely to diffuse through the GFRP rebar and degrade their component materials. In particular, glass fibers are prone to hydrolytic degradation according to the etching process reported in Equation (1) [1]. Alkaline environments also promote the concentration and growth of hydration products (such as calcium hydroxide) at the surface of glass and between individual glass filaments, which contributes to weaken the fiber/matrix interface [2]. These degradations can potentially lead to a reduction in the mechanical properties of the GFRP rebars and/or alter the bond between the rebar and the surrounding concrete.

(1) $$-\begin{array}{c} |\\ \mathrm{Si}\\ | \end{array}-O-\begin{array}{c} |\\ \mathrm{Si}\\ | \end{array}-+{\mathrm{OH}}^{-}\;\to -\begin{array}{c} |\\ \mathrm{Si}\\ | \end{array}\mathrm{OH}+-\begin{array}{c} |\\ \mathrm{Si}\\ | \end{array}O^{-}$$

Several studies are available in the literature regarding the durability of GFRP bars subjected to alkaline environments. Experimental programs are most often based on accelerated aging tests wherein GFRP rebars are exposed to different alkaline conditions (immersion in synthetic concrete pore solutions or embedment in moist cement mortar, for instance) [1,3,4,5,6,7,8,9,10,11,12,13,14,15,16]. Exposure is usually performed at elevated temperature in order to accelerate the diffusion rate of alkali ions, but it is generally recommended to stay below 60 °C to prevent side reactions that are not encountered under actual service conditions [17]. Mechanical characterizations (by tensile, short-beam, or pull-out tests) are then performed on aged specimens to assess the possible losses of mechanical properties of the bars or the changes in bond properties at the bar/concrete interface over exposure [1,18,19,20,21,22]. To go further and make extrapolations on the long-term behavior, the results of accelerated aging tests are used in predictive models based on the Arrhenius law [1,5,6,11,12,13,16,23,24,25]. The main assumption of these models is to consider that the material degradation results from a single predominant mechanism (such as the alkaline attack reported in Equation (1)) and exhibits temperature-dependent kinetics.

In the previous studies, the direct immersion of the GFRP rebars in alkaline solutions generally resulted in the significant degradation of the tensile strength over relatively limited time. For instance, Chen et al. reported strength losses up to 80% in the case of GFRP rebars aged for 240 days in a solution of pH 12.7 at a temperature of 60 °C [3]. However, this accelerated aging condition can be considered an excessively harsh environment for GFRP rebars, since field investigations report only minor degradations on GFRP reinforcements extracted from real RC structures after service periods of 5 to 20 years [26,27,28,29,30,31]. Besides, the type of polymer matrix was also found to have a large influence on the durability of GFRP rebars under direct immersion [12,32]. Vinylester matrices generally confer better resistance against chemical attack than epoxy matrices. In addition, polyester matrices exhibit high sensitivity to alkaline hydrolysis and are therefore unsuitable for permanent reinforcement applications.

Accelerated aging of GFRP rebars in moist cement mortar or in moist concrete has only been investigated by few authors [1,6,11,13] and resulted in much more limited losses of tensile strength compared to direct immersion in alkaline solutions, due to the restricted mobility of alkali ions. For instance, Robert et al. [11] reported a strength loss of 16% after 8 months exposure in moist concrete at 50 °C, and they also extrapolated a long-term loss of 20% over a period of 100 years in moist concrete at 6 °C using the predictive Arrhenius approach. According to these authors, this aging protocol in moist concrete is more representative of the actual service conditions of GFRPs in RC structures. 

Regarding the influence of accelerated aging on the bond behavior between GFRP rebars and concrete, fewer studies are available in the literature [1,18,19,20,21,22]. A comprehensive review of existing durability studies based on pull-out testing has been recently proposed by Gravina et al. [33]. It shows a large dispersion in the results of the literature, attributed to the variability of the studied materials and the absence of standard accelerated aging protocols for pull-out specimens. In general, most authors observed losses in bond strength of 8 to 15% for GFRP/concrete specimens after several months of immersion in alkaline solution or in water at 50 or 60 °C [1,20]. Some other reported bond strength losses of up to 40% in the same condition [22], and even up to 66% for pull-out specimens immersed at 80 °C [18]. Regarding the specific effect of temperature, Masmoudi et al. [21] described bond losses of up to 14% on pull-out test specimens aged for 240 days in air at 80 °C.

In this context, the objective of the present study is to provide additional experimental data regarding the accelerated aging behaviour of GFRP rebars and GFRP/concrete interfaces under alkaline environments. Glass/vinylester rebars of nominal diameter 12.7 mm were exclusively considered in this work. An accelerated aging program was conducted on these rebars subjected to direct immersion in an alkaline solution and on the same type of rebars previously embedded in a concrete cylinder before immersion in the solution (this specific condition will subsequently be denoted indirect immersion). The objective of comparing these two conditions (direct/indirect immersion) is to check that the degradation kinetics is slowed down by reducing the mobility of hydroxyl ions thanks to the intermediate concrete coating. Pull-out specimens were also exposed to the alkaline solution in order to study the durability of the rebar/concrete interface. Exposures were carried-out at different temperatures (20 °C, 40 °C and 60 °C) in order to vary the acceleration factor of the degradation kinetics and for different periods up to 240 days. A total of 130 samples was considered in this durability program. Mechanical characterization was performed at selected aging times (0, 120 and 240 days) by tensile, short-beam and pull-out tests (depending on the type of sample) in order to evaluate the residual properties. An Arrhenius approach was proposed to predict the long-term evolution of the tensile strength in the case of rebars subjected to direct immersion. Besides, physico-chemical characterizations were also carried out by various techniques (SEM, energy-dispersive X-ray analyses, FTIR and DSC) to detect the possible microstructural/chemical degradations of the GFRP rebars over aging and compare the effects of direct/indirect immersion protocols.

## 2. Materials and Methods

### 2.1. Samples

The rebars considered in this study were commercially available sand-coated glass/vinylester rods manufactured by Pultrall Inc. Company (Thetford Mines, QC, Canada) under the brand V-ROD. All GFRP rebars had a nominal diameter of 12.7 mm. As previously mentioned, in the framework of the accelerated aging program, some rebars were directly immersed in an alkaline solution, while others were embedded in concrete before being immersed in the same alkaline solution (indirect immersion). One of the objectives of this study was to compare the effects of direct and indirect exposures.

Additionally, pull-out specimens were also aged in the alkaline solution to evaluate the evolution of the bond properties at the rebar/concrete interface.

#### 2.1.1. Samples for Direct Immersion 

**Figure 1:**
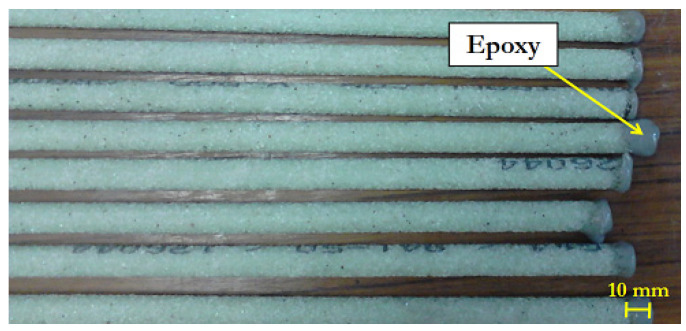
Rebars intended for direct immersion in the alkaline solution.

In order to evaluate the impact of direct immersion on the longitudinal tensile strength and elastic modulus of the GRFP rebars, samples of length 1.20 m were immersed in the alkaline solution (the composition of the solution is specified in Section 2.2). Before immersion, the edges of the rebars were locally sealed with an epoxy resin in order to prevent the capillary diffusion of moisture and alkali ions from the cut sections of the rebars (Figure 1).

Shorter rebars (32 cm long) were also prepared according to the same protocol. After aging under direct immersion, these bars were cut into three sections of 10 cm, which were then tested by short-beam test in order to evaluate their residual interlaminar shear strength (ILSS). The remaining 2 cm were used for microstructural observations and physico-chemical analyses (see Section 2.3.2, Section 2.3.3 and Section 2.3.4.

#### 2.1.2. Samples Embedded in Concrete for Indirect Immersion

**Figure 2:**
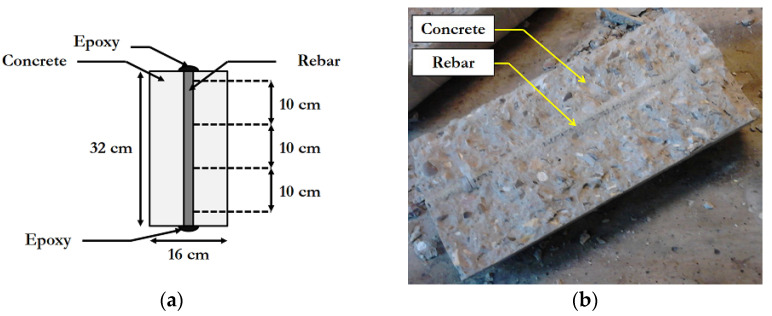
ILSS test specimens for indirect immersion: (**a**) geometry of specimens and (**b**) picture of a specimen after the splitting of surrounding concrete.

Some 32 cm long rebars were embedded in concrete cylinders with 16 cm diameter, before being immersed in the alkaline solution. The geometry of these specimens is shown in Figure 2a. Before immersion, the edges of the rebars were again locally sealed with an epoxy resin to prevent the penetration of alkalis through the cut sections.

All specimens were made from a single batch of normal strength concrete, formulated with aggregates with a 22 mm maximum size. The compressive strength of concrete was determined on three cylindrical specimens (∅ 16 cm × 32 cm) after 28 days of cure out of water. These tests provided a mean compressive strength of 29.4 ± 0.4 MPa. More details regarding the concrete formulation and the compressive tests are available in [34].

After 28 days of cure, a part of the embedded GFRP specimens was immersed in the alkaline solution at different temperatures (20 °C, 40 °C and 60 °C). This configuration allows the studying of the effect of alkali diffusion from the solution to the core GFRP rebar through the porous structure of the surrounding concrete cylinder, while ensuring an ionic balance between the solution contained in the pores and the external solution. The mobility of alkali ions is expected to be significantly lower than in the case of direct immersion of GFRP rebars in the solution. Nevertheless, it was chosen to use a normal-strength concrete, which is generally more porous than high strength concrete. This choice was deliberately made to preserve a certain diffusion of the alkalis in the concrete pore, so that the conditions of the study remain sufficiently conservative.

Another portion of the specimens was subjected to pure thermal aging at 40 °C and 60 °C in the air. Such conditioning out of the alkaline solution was considered for detecting specific effects possibly induced by the thermal solicitation (in particular, the effect of local stresses generated by differential thermal expansion between the GFRP material and concrete).

After aging, the GFRP rebars were carefully extracted from the concrete cylinders by splitting the concrete piece and then characterized by short-beam tests for evaluating their residual ILSS (this test method is detailed in Section 2.3.6). Figure 2b shows a rebar in concrete, after the splitting of the specimen.

In the same way, it might have been interesting to investigate the effect of indirect immersion on the tensile properties of GFRP rebars, for the sake of comparison with the impact of direct immersion. However, this would have required the preparation of long specimens consisting of 1.20 m-long rebars embedded in concrete cylinders. Unfortunately, this type of specimen was not included in the experimental program due to the limited storage capacity of aging chambers/tanks available at the laboratory.

#### 2.1.3. Pull-Out Specimens

**Figure 3:**
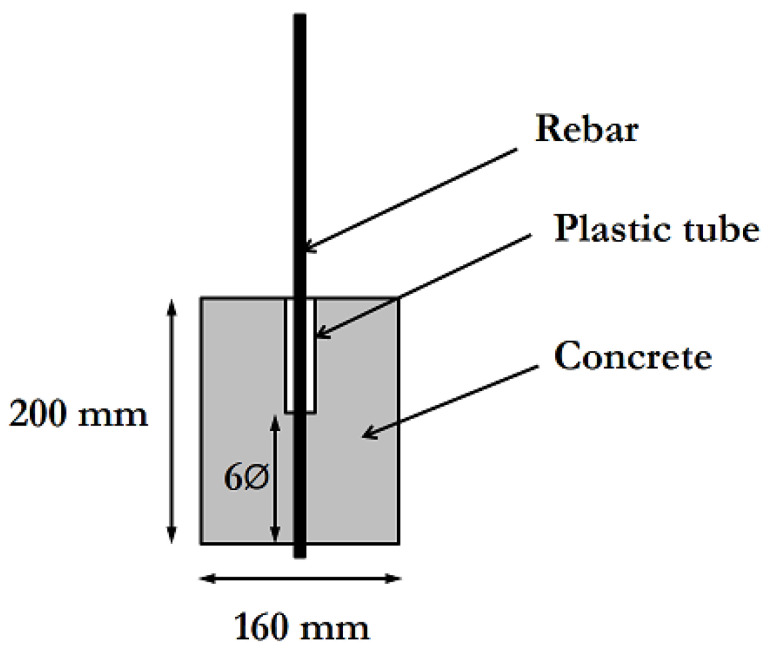
Geometry of the specimens used for pull-out tests.

In order to study the bond behavior between GFRP rebars and concrete, pull-out specimens were prepared according to the following procedure: 1.20 m-long straight rebars were partially embedded at the center of a normal strength concrete cylinder (of diameter 16 cm and height 20 cm). The specimen’s geometry is illustrated in Figure 3. Concrete was cast while the rebar was set in the vertical position. A plastic tube, called a bond breaker, was placed between the rebar and concrete at the upper side of the concrete block, in order to prevent edge effects induced by the reaction support during pull-out tests (this test protocol is detailed in Section 2.3.7). The length of the bond breaker was chosen so that the embedment length of the bar in concrete is equal to six bar diameters (Figure 3). This condition is supposed to favor preferential failure by the slippage of the rebar [35]. It is worth noting that the same batch of concrete was used for casting the pull-out specimens and for embedding the GFRPs of short-beam specimens (series exposed to indirect immersion). 

After concrete casting, pull-out specimens were stored for 28 days in the laboratory and then placed in the aging environments. A part of the specimens was immersed in the alkaline solution at the various temperatures (20 °C, 40 °C and 60 °C). The other part was again subjected to pure thermal aging in the air at 40 °C and 60 °C to assess possible thermal effects on the bond properties.

### 2.2. Accelerated Aging Protocols

**Figure 4:**
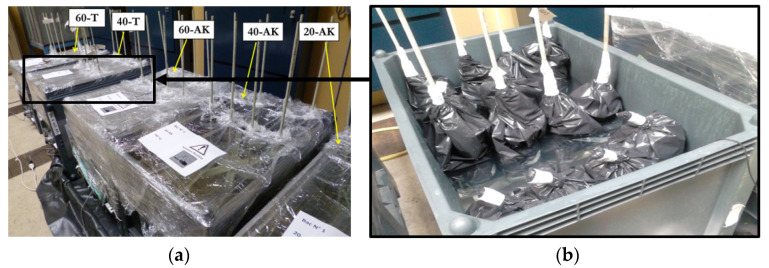
(**a**) Picture of the five thermoregulated tanks and (**b**) detail of the 40-T tank.

Five tanks were used for conditioning the samples in the different accelerated aging environments, as shown in Figure 4a. Three tanks, in which the aging environments were denoted 20-AK, 40-AK, and 60-AK, were filled with an alkaline solution and respectively maintained at room temperature (about 20 °C in the laboratory) and at constant temperatures of 40 °C and 60 °C (using heating resistances). These tanks were used both for the direct immersion of GFRP rebars and for the indirect aging of embedded specimens.

The alkaline solution was composed of 0.1 mol/L (4 g/L) of sodium hydroxide (NaOH) and 0.5 mol/L (28.05 g/L) of potassium hydroxide (KOH). This solution is commonly used at Eiffel University in the framework of durability/corrosion studies [36,37] because it is suitable for simulating the pore solution of European concretes at early age. It was preferred to the solution recommended by the ACI 440.3R [38]. The pH of the alkaline solution was measured in each tank on the first day of the aging program and after a period of 240 days as well. The corresponding values are reported in Table 1. Initial pH values were close to 13.5 regardless of the temperature of the tank, and they showed very little change after 240 days.

The two other tanks, in which the aging environments were denoted 40-T and 60-T, were filled with water and regulated at temperatures of 40 °C en 60 °C, respectively. These tanks were intended for applying pure thermal aging conditions, in order to study possible differential thermal effects between the rebars and concrete. Before being placed in these tanks, the specimens were first wrapped with hermetic plastic bags to avoid contact with water and keep them in a dry state (Figure 4b). Only embedded GFRP specimens (i.e., 16 × 32 specimens for short-beam tests and pull-out specimens) were placed in these pure thermal environments. 

Finally, the specimens were conditioned for periods of 120 and 240 days in these various environments, and then removed from the tanks in order to be tested.

### 2.3. Characterizations before and after Aging

#### 2.3.1. Monitoring of the Mass and Diameter of Aging Rebars

When rebars are immersed in an alkaline solution, water absorption may occur, possibly leading to the swelling of the polymer matrix and changes in the diameter and mass of the GFRP rod. In order to monitor these possible changes over aging, three dedicated sand-coated GFRP rebars and one uncoated rebar (a rod with a smooth finish produced by the same manufacturer) were placed in each of the tanks containing the alkaline solution environments (20-AK, 40-AK and 60-AK). The bar edges were also coated with an epoxy resin prior to immersion, to avoid moisture diffusion through the cut sections. These rebars were periodically removed from the baths and their mass was determined using a balance of precision 0.1 g, in order to assess the amount of absorbed water compared to the initial state.

**Figure 5:**
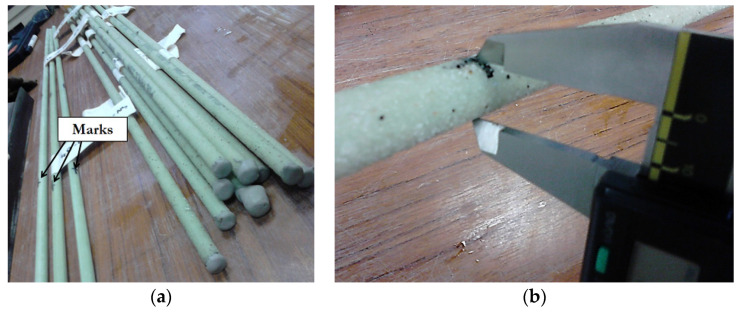
(**a**) Marked GFRP rebars used for monitoring the changes in mass/diameter over aging and (**b**) illustration of the diameter measurement on a sand-coated rebar (as a reminder, the nominal diameter of all rebars was 12.7 mm).

In addition, diameter measurements were performed with a caliper of precision 0.01 mm at fixed locations of the bars identified by marks (reticles) drawn at the surface (one mark per sand-coated rebar and 3 marks per smooth rebar). Such marked rebars are shown in Figure 5a, and an illustration of the diameter measurement on a sand-coated rebar is presented in Figure 5b.

#### 2.3.2. Microstructural Observations

Microstructural observations were made on polished cross-sections of the GFRP rebars using a Hitachi (Tokyo, Japan) S570 scanning electron microscope (SEM) operating in secondary electron mode. The objective was to detect the possible degradation of the fibers, polymer matrix or fiber/matrix interface after aging.

In addition, analysis of the surface elemental composition was performed using a Quantax energy-dispersive X-ray (EDX) probe from Bruker Corporation (Billerica, MA, USA). This technique enables the mapping of the spatial distribution of a given chemical element at the surface of the sample. It was used to detect possible concentrations of K or Na elements in the aged rebars that would clearly evidence the diffusion of alkali ions into the sample during aging.

#### 2.3.3. Differential Scanning Calorimetry

Samples taken from the surface and the core of aged rebars were analyzed by differential scanning calorimetry (DSC), using a DSC Q100 apparatus from TA Instruments (New Castle, DE, USA). Analyses were carried out according to the NF EN ISO 11357-2 standard [39]. The samples were subjected to two successive ramps of temperature between 0 and 200 °C with a heating rate of 10 °C/min and under inert nitrogen environment. The collected thermograms allowed the determination of the glass transition temperatures of the vinylester matrix at the first and second runs, denoted *T_g_*(1) and *T_g_*(2) respectively. The objective was to detect possible changes in these glass transition temperatures during aging that would reveal microstructural modification or chemical alteration of the vinylester network [40].

#### 2.3.4. Fourier Transform Infrared Spectrometry

Fourier transform infrared (FTIR) analyses were performed using a Nicolet 380 spectrometer from Thermo Fisher Scientific (Waltham, MA, USA). This technique allows the detection of the characteristic vibrations of chemical bonds and therefore, to highlight the different functions/molecular groups that compose the chemical structure of a material. These analyses were carried out in attenuated total reflectance (ATR) mode on samples taken near the rebar surface. Spectra were collected in the wavelength range 4000–400 cm^−1^, with a resolution of 4 cm^−1^ and an accumulation of 32 spectra. The objective was to detect any changes in the chemical structure of the vinylester matrix after aging.

#### 2.3.5. Tensile Tests

After aging by direct immersion in the alkaline solution at 20 °C, 40 °C and 60 °C, the 1.20-m-long rebars were characterized in tension according to the ASTM D7205 standard [41] in order to determine the residual elastic modulus and tensile strength. 

Before testing, a specific anchoring device was sealed on each end of the rebars. This device consists of a steel tube filled with an expansive mortar, allowing a more uniform distribution of the radial stresses in the rebar at the time of testing and thus, avoiding premature failure in the anchoring areas of the press jaws. Tests were carried out using a 250 kN universal testing machine from Instron (Norwood, MA, USA). Loading was controlled by the upper cross-beam displacement with a displacement rate of 10 mm/min. During the test, the rebar deformation was locally measured by a linear variable differential transformer (LVDT) type extensometer. More details on this experimental setup can be found in [34].

#### 2.3.6. Short-Beam Tests

After both aging by direct and indirect immersion, the samples intended for interlaminar shear characterization were tested by the short-beam method, according to ASTM D4475 standard [42].

**Figure 6:**
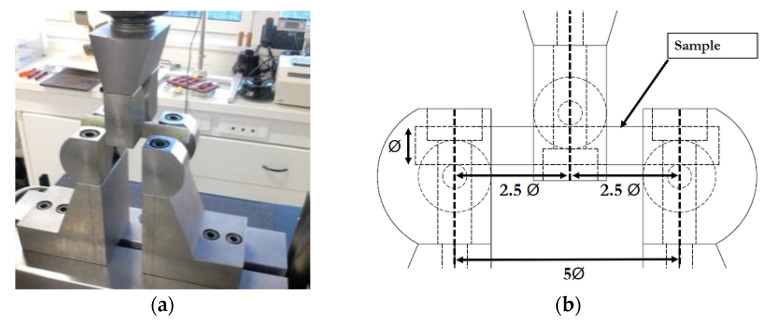
Short-beam test: (**a**) picture and (**b**) detailed scheme of the test configuration (dimensions are in mm).

Tests were carried out on 100 mm-long rebar samples. Three identical samples were tested for each aging condition and aging time. To perform these tests, a specific frame (Figure 6) was manufactured at the laboratory and installed on a universal testing machine of capacity 50 kN, model 5969 from Instron (Norwood, MA, USA). The specificity of this frame relies on the geometric shape of the supports, which is designed to ensure quasi-punctual contact and avoid any slip of the circular cross-section of the sample. The distance between the two lower supports was set to 5 times the rebar diameter Ø (Figure 6b). The test was controlled by the displacement of the upper support at a rate of 1 mm/min. The apparent interlaminar shear strength (ILSS) of the tested rebar was deduced from the ultimate load *F* by the relation ILSS = 0.849 *F*/Ø^2^ [42].

#### 2.3.7. Bond Tests

**Figure 7:**
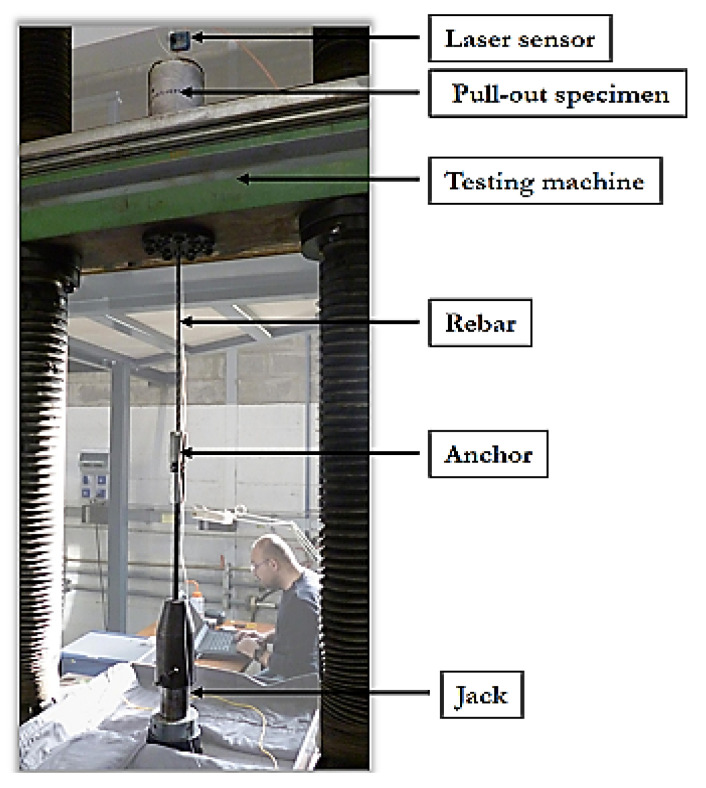
Picture of the pull-out test setup.

The specimens dedicated to bond characterizations were tested by the pull-out method according to the ACI 440.3R-04 guidelines [38], using a 350 kN Losen testing machine. The test setup is shown in Figure 7. Specimens were installed on the drilled horizontal crosshead beam of the machine, with the rebar passing through the beam hole. The rebar is loaded using a jack, via a wedge-type anchor. The loading is controlled by the displacement of the jack at a speed of 1.2 mm/min.

At the end of the test, the bond strength *τ_b_* can be determined as the ratio of the maximum tensile force *F* exerted on the loaded end to the surface area of the rebar embedded in concrete. It is expressed in the following form:

(2) $$\tau_{b}=\frac{F}{\pi \phi L}$$

In this expression, ϕ is the rebar diameter, and *L* is the embedment length in concrete. As a reminder, the frame is embedded over a length equal to six times its diameter (see Figure 3). Further details regarding the program of pull-out tests conducted in the framework of this study are available in [34,43,44].

### 2.4. Summary of the Experimental Programme

**Figure 8:**
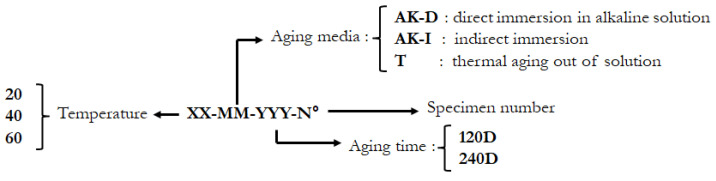
Nomenclature of the different aging protocols.

The 130 specimens from this study are referenced in Table 2. This list includes rebar specimens dedicated to the monitoring of the mass/diameter, which are identified by the extension –MASS-S for sand-coated rebars or –MASS-NS for non-coated rebars (smooth finish). The nomenclature of the different aging conditions is explained in Figure 8.

In addition to the specimens listed in Table 2, 3 cylindrical concrete samples (diameter 16 cm, height 32 cm) were also placed in each aging tank. Compression tests were carried out on these cylinders after 240 days of exposure in order to assess possible changes in the concrete properties after aging.

## 3. Results and Discussions

### 3.1. Microstructural Observations

**Figure 9:**
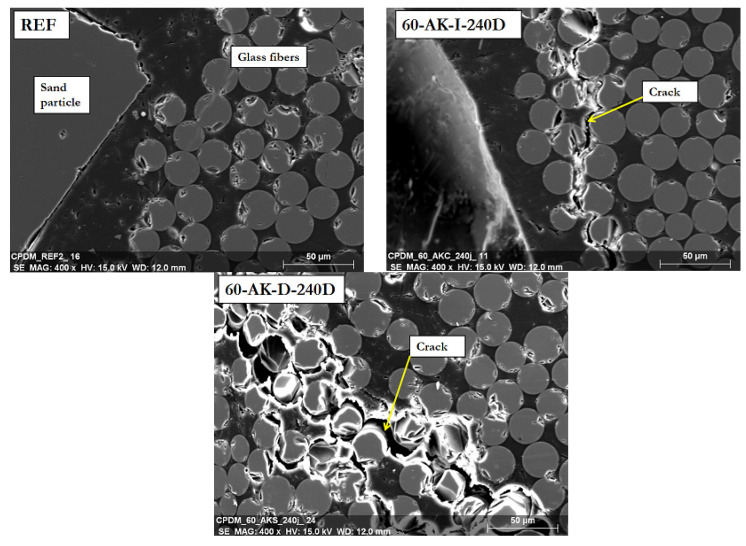
SEM images of the cross section of reference and aged GFRP rebars, at locations close to the external sand coating layer.

Figure 9 displays typical SEM images showing the periphery of the cross-sections of GFRP rebars after 240 days at 60 °C in AK-D and AK-I conditions. The picture of a reference rebar is also provided for comparison. One can observe many cracks on the 60-AK-D-240D sample but only exclusively near the external sand coating layer and not in the central part of the bar cross-section. Some cracks are also detected on the 60-AK-I-240D samples but to a much lower extent. Since the rebars have not been mechanically stressed during or after aging, these cracks are therefore either directly related to aging-induced degradation or due to the polishing process on samples used for SEM observation. In both cases, these cracks reveal an embrittlement of the fibers and the fiber/matrix interface induced by aging at the outermost region of the rebars, since an identical surface preparation did not damage the reference samples. Such embrittlement process is more severe in the case of direct immersion in the alkaline solution than for the indirect aging condition.

**Figure 10:**
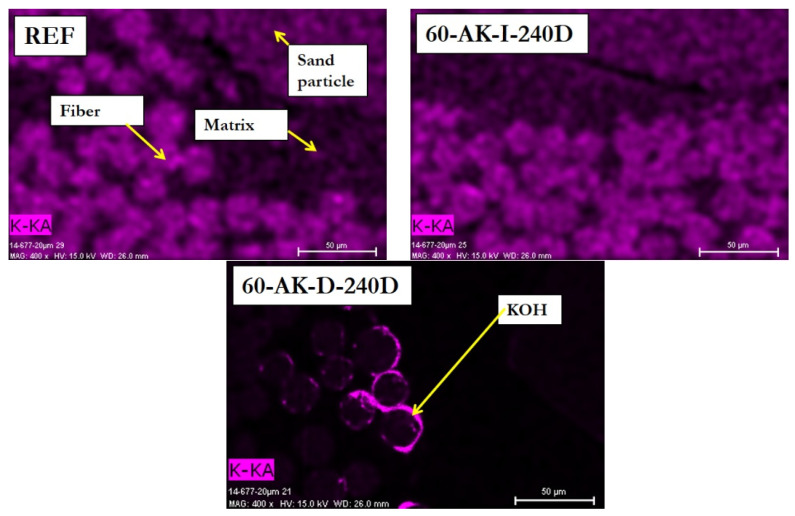
EDX mapping of K over cross-sections of the reference and aged GFRP rebars, at locations close to the external sand coating layer.

Complementary EDX analyses were carried out at the surface of the same polished samples, in order to monitor the possible migration of alkalis from the aging solution to the inside of the rebars. Figure 10 displays the typical mappings of the spatial distribution of potassium element (K) over the analyzed cross-sections. This element is a good marker of the alkaline solution, which is mainly composed of potassium hydroxide (KOH).

For the 60-AK-D-240D sample, the concentration of K can be observed at fiber/matrix interfaces close to the external coating of the rebar. In the reference sample and in the 60-AK-I-240D rebar, the distribution of K is much more diffuse and probably relates to some potassium initially present in the composition of the materials (especially in the glass fibers). This result suggests that the substantial diffusion of alkalis may occur within GFRP rebars subjected to direct immersion, but it remains restricted to the outermost layer of the bar.

To conclude, SEM images and EDX analyses provide evidence for the greater severity of the aging protocol based on the direct immersion of GFRP rebars in the alkaline solution compared to the indirect immersion involving an intermediate concrete cover. Note that rather similar conclusions were drawn by several authors when comparing direct immersion in an alkaline solution to indirect immersion in water (GFRP bars aged in moist concrete) [1,6,11,12].

### 3.2. Mass and Diameter Evolutions

Table 3 reports the mass gains determined for sand-coated (S) and non-sand coated (NS) rebars over 120 days of immersion in the alkaline solution at 20 °C, 40 °C and 60 °C. Overall, very little mass variations were obtained for all specimens, showing very limited absorption of the solution by the GFRP rebars. Nevertheless, one may notice slightly higher mass gains for sand-coated bars compared to smooth ones. This difference suggests that the limited absorption phenomenon mainly takes place in the sand coating layer.

Table 4 presents the diameter variations measured on sand-coated and non-coated rebars subjected to direct immersion over a period of 120 days. On the overall, results show little variations for all specimens, despite the large experimental dispersion. In the case of sand-coated rebars, the trend even suggests a reduction in diameter. However, it should be recalled that measurements were made with a caliper. This technique was not very adequate for sand-coated bars, as some sand particles detached from the bars during handling/measurement operations, which may have affected the results.

### 3.3. Physico-Chemical Properties of the Polymer Matrix

#### 3.3.1. Glass Transition Temperature

DSC analyses were performed on small samples (a few mg) taken from the surface (just beneath the sand coating layer) of GFRP rebars aged for 240 days by direct immersion in the alkaline solution (AK-D) at 20 °C, 40 °C and 60 °C and on samples taken from the surface of unaged reference rebars as well (REF). Analyses were also carried out on samples taken from the core of GFRP rebars, in the case of reference and 60-AK-D specimens. The glass transition temperatures (*T_g_*) of the vinylester matrix determined from DSC thermograms are reported in Table 5.

Very little variation of the glass transition temperature is observed over aging. This is true for both values determined at the first run (*T_g_* (1)) and at the second run (*T_g_* (2)), regardless of the location of the sample (surface or core). The vinylester matrix of these GFRPs seems therefore little affected by direct immersion conditions and exhibits good chemical stability under alkaline environments.

Besides, one can notice a difference between the values of *T_g_* (1) and *T_g_* (2) for a given sample (the value obtained at the second run is always about 10 to 15 °C higher than that at the first run). This is verified for both the reference sample and the aged rebars. It shows that the vinylester matrix has not been fully cured during the manufacturing process and that some post-curing effect takes place during the first DSC run, leading to an increase in the glass transition temperature at the second run. 

#### 3.3.2. FTIR Analyses

**Figure 11:**
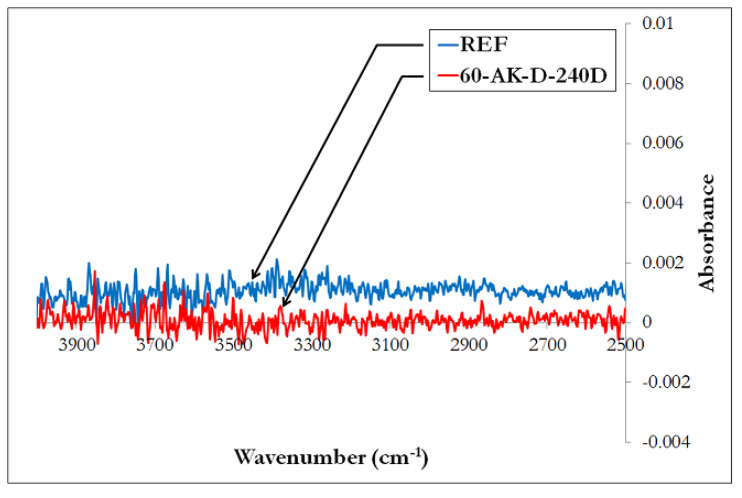
FTIR spectra in the region of 2500–4000 cm^−1^ from a reference sample (REF) and from a sample aged in the alkaline solution at 60 °C for 240 days (60-AK-D-240D).

Figure 11 displays the typical FTIR spectra recorded for unaged reference samples and for samples aged by direct immersion in the alkaline solution at 60 °C for 240 days (60-AK-D-240D). On these spectra, the region between 3300 and 3600 cm^−1^ is of great interest as it contains the vibration bands of hydroxyl groups (OH) [11]. In this region, the comparison of spectra from the reference and aged samples does not reveal any significant change induced by alkaline aging. In particular, no additional hydroxyl groups is detected, hence there is no evidence of hydrolytic degradation of the polymer matrix. This result is consistent with previous DSC analyses and confirms that the vinylester matrix used in these GFRP rebars exhibits good resistance to alkaline environments. 

### 3.4. Tensile Behavior of the Rebars after Direct Immersion

#### 3.4.1. Longitudinal Tensile Strength and Elastic Modulus

**Figure 12:**
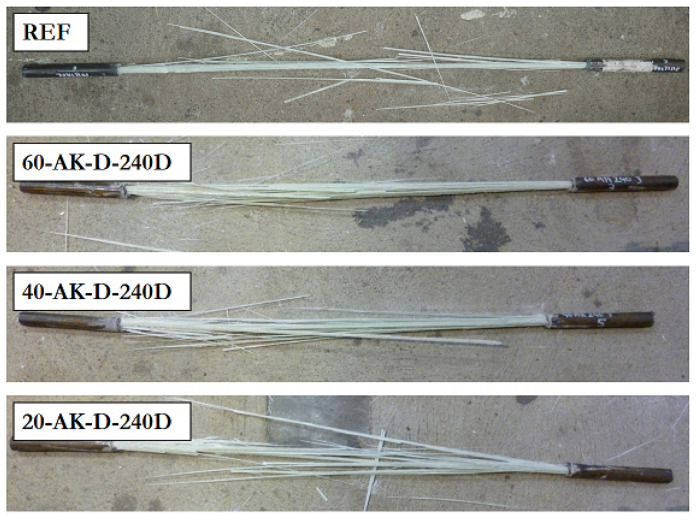
Typical failure modes after tensile tests on aged GFRP rebars and reference samples.

All GFRP rebars showed brittle failure during a tensile test. Figure 12 presents the typical tensile failure modes obtained in the case of GFRP rebars exposed to direct immersion in the alkaline solution for 240 days (AK-D-240D) at different temperatures and for the unaged reference bar as well. Visual observation does not reveal any change in failure mode resulting from aging.

**Figure 13:**
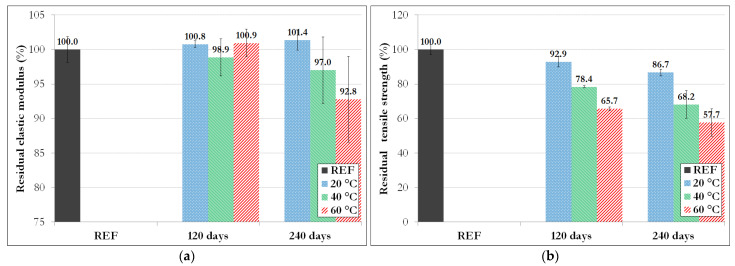
Residual tensile properties of GFRP rebars subjected to direct immersion in the alkaline: (**a**) elastic modulus and (**b**) tensile strength.

The elastic modulus and tensile strength determined for the reference specimens and the aged GFRP rebars are reported in Table 6 and summarized in the graphs of Figure 13.

Experimental results reveal only small variations in the elastic modulus after aging (with a maximum loss of 7% after 240 days at 60 °C). Conversely, a substantial decrease in the tensile strength is observed after aging. In particular, one can notice (i) a gradual decrease in resistance over time and (ii), at a given time, a drop of resistance when raising the aging temperature (strength loss up to 42% after 240 days at 60 °C). 

These trends suggest that significant degradation of the material takes place over prolonged direct immersion of GFRP rebars in the alkaline solution, in accordance with previous SEM observations. They also corroborate observations made by other authors [1,5,7], who reported significant reductions in the tensile strength of GFRP bars after direct immersion in alkaline solutions.

Nevertheless, one must remind that this accelerated condition is much more severe than the actual exposition in concrete structures [1,6,11,12]. As already mentioned in the Introduction section, Robert et al. [11] showed that GFRP rebars embedded in moist concrete at 50 °C lost about 16% of their tensile strength after 250 days exposure, whereas for a similar duration (240 days), our specimens exposed to direct immersion at 40 °C and 60 °C experienced much higher strength losses of 31% and 42%, respectively.

#### 3.4.2. Long-Term Prediction Based on the Arrhenius Approach

In this paragraph, we propose to quantify the acceleration effect produced by temperature on the degradation kinetics of GFRP rebars exposed to direct immersion in the alkaline solution. This approach is based on the Arrhenius law, which has been widely used in the literature for predicting the degradation behavior of composite rebars [1,5,6,12,13,16,23,24,25]. It is assumed that a single chemical process is responsible for the material degradation (i.e., the hydrolysis of siloxane bridges in glass fibers). Assuming that the reaction proceeds under a constant temperature *T*, the reaction kinetics is characterized by the rate coefficient *k*, which can be expressed in the following form, according to Arrhenius law:

(3) $$k=A\exp\left(-\frac{E_{a}}{RT}\right)$$

where *k* is the reaction rate coefficient related to the rate of degradation (homogeneous to the inverse of time); *E_a_* is the activation energy of the reaction (in J·mol^−1^); *R* is the ideal gas constant (in J·mol^−1^·K^−1^); *T* is the absolute temperature (in K), and *A* is a pre-exponential factor.

Let us consider two isothermal tests, test 0 and test 1, carried out respectively at temperatures *T*_0_ and *T*_1_, during periods *t*_0_ and *t*_1_ and having led to degradation rates *k*_0_ and *k*_1_. For an equivalent level of degradation, the ratio between the time *t*_0_ necessary to achieve this degradation level in test 0 and the time *t*_1_ necessary in test 1 is called the time shift factor (TSF). It can be written in the following form:

(4) $$TSF_{T_{1}/T_{0}}=\frac{t_{0}}{t_{1}}=\frac{k_{1}}{k_{0}}=\exp\left[\frac{E_{a}}{R}\left(\frac{1}{T_{0}}-\frac{1}{T_{1}}\right)\right]$$

Let us now denote by *R*(*t*) the residual tensile strength of the rebars at any time *t* during the degradation process. We propose to describe the residual tensile strength evolution *R*(*t*), expressed as a percentage of the initial resistance value, in the following form:

(5) R(t)=100exp(−t/τ)

where *τ* is the characteristic degradation time, which depends on the temperature.

**Figure 14:**
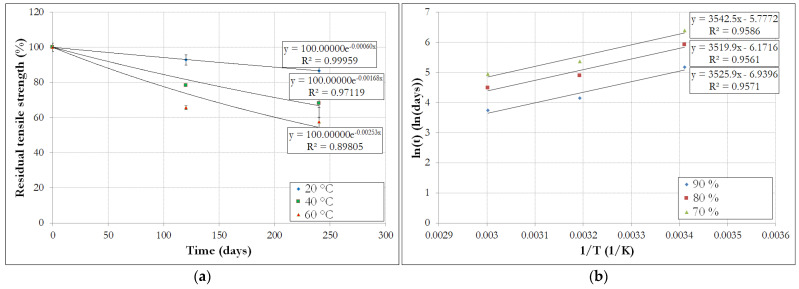
(**a**) Residual tensile strengths of GRP bars as a function of direct aging time and (**b**) Arrhenius lines for different retention rates of the tensile strength.

From experimental data, it is possible to trace the evolution of the residual tensile strength of rebars (in %) as a function of time for each aging temperature (see Figure 14). Although only three values of residual strength (0 days, 120 days and 240 days) are available for each temperature in our present study, exponential regressions were performed using Equation (5) on these data, in order to determine the three *τ* coefficients for the three test temperatures considered in this study (20 °C, 40 °C and 60 °C). The results of these identifications are displayed in Figure 14a. In order to increase the robustness of the data fitting, it would be necessary to perform other measurements at additional aging times. Unfortunately, this was not possible in the present study due to time constraints.

Figure 14b presents, from the identified curves *R*(*t*) at different temperatures, the natural logarithm of the reaction time necessary to reach residual tensile strength values of 90%, 80% and 70%, versus the inverse of the absolute temperature. Linear regressions of these data series, also called Arrhenius lines, make it possible to estimate the quantity *E_a_*/*R,* which is equal to the slope of the lines. Indeed, from Equation (3), we deduce:

(6) $$\ln\left(\frac{1}{k}\right)=\frac{E_{a}}{R}\frac{1}{T}-\ln\left(A\right)$$

It is worth noting that the three Arrhenius lines displayed on Figure 14b are almost parallel, which supports the initial hypothesis of a single degradation reaction mechanism or at least of a dominant mechanism.

After the identification of the slopes of Arrhenius lines in Figure 14b, it can be estimated that *E_a_*/*R* = 3500 K. Considering a reference temperature of 20 °C, TSF can then be determined at 40 °C and 60 °C using Equation (4), and their values are reported in Table 7.

**Figure 15:**
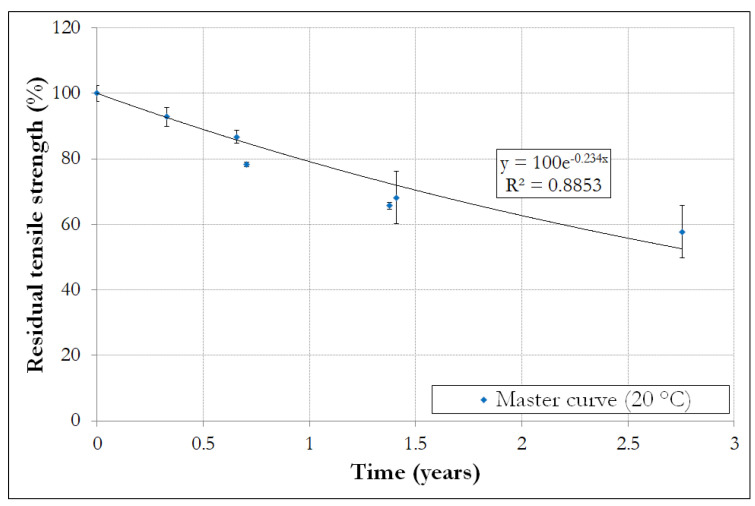
Master curve (residual tensile strength versus aging time) at a reference temperature of 20 °C.

Finally, knowledge of the temperature shift factors enable the construction of the master curve describing the long-term evolution of the residual tensile strength at the reference temperature of 20 °C, as shown in Figure 15.

This master curve predicts a large degradation of the tensile strength of about 40% after 2.5 years of exposure. Similar predictions were reported by [3] on glass/vinylester FRP rebars aged in an alkaline solution. However, it must be recalled that this direct aging condition is very harsh and provides extremely pessimistic results.

Robert et al. [11], who investigated the aging behavior of similar GFRP rebars in moist concrete at 20 °C, 40 °C and 50 °C for periods of up to 8 months, also proposed long-term extrapolation based on the Arrhenius approach. In these conditions, they predicted tensile strength losses of only 20% and 25% after, respectively, 100 and 200 years at a reference temperature of 6 °C. This trend is more in line with field investigations conducted on real structures, which do not report significant degradation of the GFRP rebars after 5 to 20 years of service [26,27,28,29,30,31].

The notable divergence between tendencies resulting from the two accelerated aging methods (moist concrete versus direct immersion in alkaline solution) stress the need to carry out further comparative studies between real aging/accelerated aging, in order to be able to recommend a representative accelerated aging protocol in the future.

### 3.5. Interlaminar Shear Strength of the Rebars after Direct and Indirect Aging

**Figure 16:**
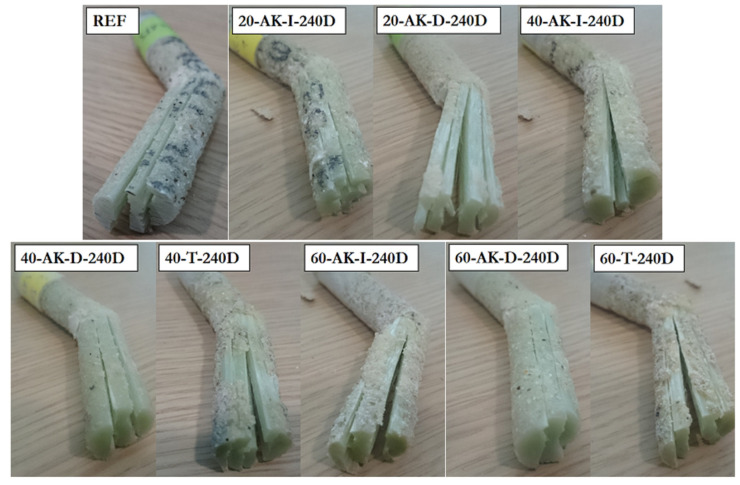
Failure modes observed after short-beam tests on reference and aged GFRP rebars.

Short-beam tests were performed on reference samples and on GFRP rebars exposed for 240 days to direct (AK-D) and indirect (AK-I) immersion in the alkaline solution at different temperatures and to pure thermal aging (T) as well. Figure 16 shows that the failure mode was identical for all specimens and was initiated by delamination at the neutral axis of the GFRP bars.

**Figure 17:**
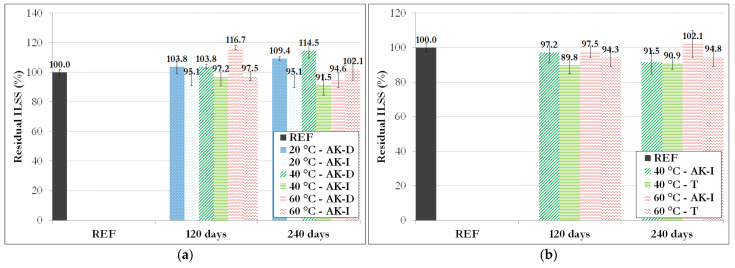
Residual interlaminar shear strength of GFRP rebars after aging in different environments: (**a**) AK-D versus AK-I and (**b**) AK-I versus T.

ILSS values determined on the various specimens are referenced in Table 8 and summarized in Figure 17. 

At first, the results show that accelerated aging does not induce a significant decrease in the ILSS of GFRP rebars, whatever the type of accelerated environment. With regard to rebars subjected to direct immersion in the alkaline solution (AK-D), it is even possible to note slight increases in ILSS (except for the 60-AK-D-240D samples). The trend observed here is therefore very different from that previously described for the tensile strength. Note that the failure mechanisms of these two tests are very different, because the ILSS value relates more to the properties of the matrix and fiber/matrix interface, while the longitudinal tensile strength is mainly governed by the tensile properties of glass fibers. The relative stability of the ILSS of aged bars would therefore confirm the good chemical resistance of the vinylester matrix that had already been demonstrated previously by DSC and FTIR analyses and also the good resistance of the fiber/matrix interface (at least in the core specimen, as SEM observations revealed some cracks in the outermost region).

In addition, for a given temperature and exposure time, rebars exposed to direct immersion (AK-D) exhibit slightly higher ILSS values compared to rebars exposed to indirect immersion (AK-I), and some values may even be greater than the initial ILSS (up to a 17% increase for 60-AK-D-120D rebars). Because this property (ILSS) related the chemical resistance of the matrix and the fiber/matrix interface, direct immersion in the alkaline solution does not therefore represent a particularly penalizing condition.

The results also highlight higher ILSS values for the rebars subjected to the AK-I environment compared to pure thermal aging out of solution (T). This phenomenon could result from a possible confinement effect of the fibers in the AK-I condition, driven by the swelling of the polymer matrix due to the absorption of some interstitial solution. Such a confinement effect would be beneficial to the fiber/matrix load transfer and hence to the ILSS. Finally, the overall higher ILSS values obtained for rebars aged in direct immersion (AK-D) compared to those aged in the two other environments could result from a greater diffusion of the solution in these AK-D samples, hence a larger swelling phenomenon of the polymer matrix and a stronger confinement effect of the fibers. However, this hypothesis remains uncertain, as the monitoring of the mass and diameter of GFRP rebars did not reveal very significant evolution.

The results presented in this section are quite remarkable, as in previous work by Chen et al. [1], focused on the aging behavior of glass/vinylester FRP rebars in alkaline solution, slight losses in ILSS were reported (up to 8% after immersion at 60 °C for 45 days). Higher losses were even noted by Kim et al. [10] (up to 17% and 24% for two different types of glass/vinylester FRP rebars immersed at 40 °C for 60 days).

The vinylester resin of GFRP rebars used in the present study therefore appears to be very stable in alkaline environments.

### 3.6. Bond Properties of the GFRP Rebars with Concrete

#### 3.6.1. Evolution of the Concrete Properties over Aging

Bond properties of GFRP rebars with concrete are closely related to the properties of the host concrete. To explain the possible evolution of the bond properties during aging, it is thus necessary to monitor the evolutions of the concrete properties in the same aging conditions as well. In this line, concrete cylinders (diameter 16 cm, height 32 cm) were stored in the various aging environments (immersion in the alkaline solution and pure thermal aging in the air at different temperatures) in order to be tested in compression after 240 days of exposure. 

**Figure 18:**
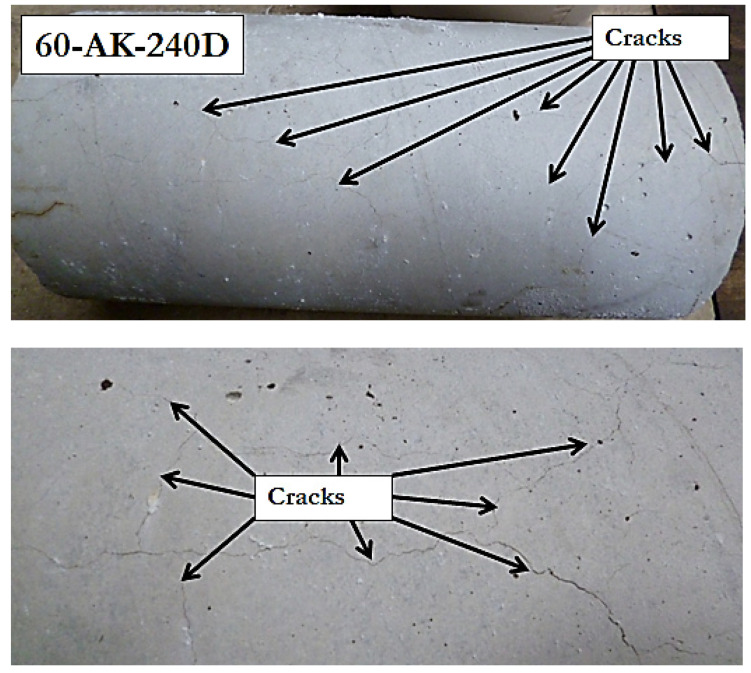
Typical surface aspect of 60-AK-240D concrete cylinders.

Results of the compression tests are reported in Table 9 for both aged specimens and reference samples. Overall, one can notice an increase in the compressive strength after aging, which accounts for a notable post-curing effect related to the progress of cement hydration reactions with time. As an exception, the 60-AK-240D specimens were the only one that exhibited a loss in compressive strength, which nevertheless remained limited (about 10%). This phenomenon was correlated to the development of multidirectional cracks regularly distributed at the surface of the cylinders, as illustrated in Figure 18. These cracks suggest a possible internal swelling reaction of the concrete, but further investigations would be necessary to identify more precisely the origin of this phenomenon.

#### 3.6.2. Results of Pull-Out Tests

**Figure 19:**
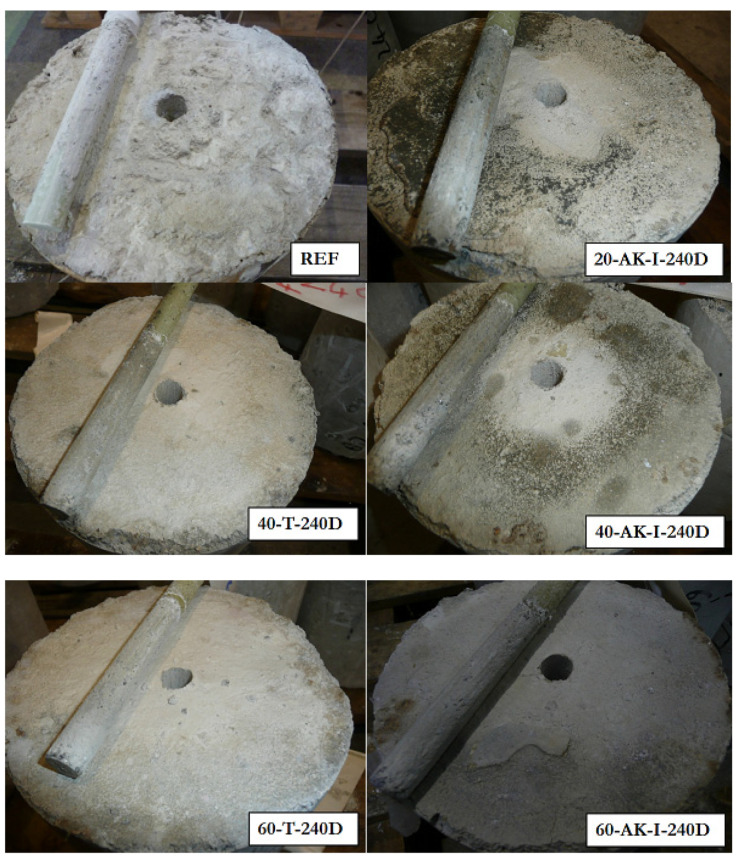
Typical failure modes observed after pull-out tests on reference and aged specimens.

Pull-out tests were then carried out on specimens aged for 240 days in the different environments, and the typical failure modes are illustrated in Figure 19. Overall, there is very little difference between the failure modes before and after aging, regardless of the type of aging environment. Failure always occurs at the interface between concrete and the sand coating of the GFRP rebar.

**Figure 20:**
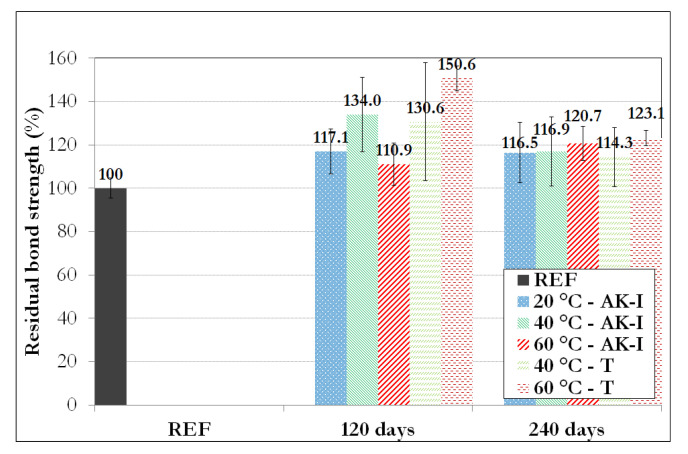
Residual bond strengths of pull-out specimens exposed to the different aging environments for different periods.

Bond strength values determined on reference and aged pull-out specimens are reported in Table 10 and summarized in Figure 20. 

Globally, an initial increase in bond strength is observed over the first 120 days of exposure, regardless of the type of aging environment, followed by a decreasing trend between 120 days and 240 days. The bond strength values at 240 days of exposure still remain higher than that of the reference specimens. Note that experimental dispersion was rather large in the case of aged samples, which made comparisons difficult.

The improvement in the concrete properties over aging (as shown previously on concrete cylinders) has certainly played a beneficial role on the rebar/concrete bond as well.

In this context, the significant increase in bond strength over the first 120 days exposure may thus relate (i) to an increased shear strength of the concrete close to the concrete/rebar interface, (ii) to a confinement effect of the rebar driven by shrinkage of the post-cured concrete or (iii) to a synergy of the two phenomena. 

Nevertheless, this beneficial post-cure of concrete may have masked possible degradation mechanisms of the concrete/rebar interface after aging, at least in the first stage of exposure wherein the kinetics of the hydration reaction of concrete is still high. As this kinetics reaches a steady state, the interfacial degradation process may become predominant over the post-cure, which could explain the decreasing trend of the bond strength between 120 and 240 days of exposure. In order to validate this hypothesis and confirm the decreasing trend in the long term, further experimental programs are needed with extended periods of aging.

Regarding the 60-AK-I-240D specimens, although compression tests on concrete cylinders have revealed a loss of compressive strength, the bond strength still remains higher than that of the reference specimens. If an internal swelling reaction of concrete has actually taken place in these specimens, this reaction may have produced additional confinement of the GFRP rebars and hence, increased the bond properties.

Finally, it should be noted that with higher-performance concrete, one could also expect an initial increase in the bond strength related to the maturation of concrete. Differently, the subsequent decreasing trend might be less (or not) visible, due to the low porosity of high-performance concrete, which prevents the extensive diffusion of alkali ions.

Overall, the findings of the present study regarding the bond behavior provide more optimistic trends than results presented by other authors [1,19,20,21,22], as the bond strength of all aged specimens remained higher than that of the unaged reference.

## 4. Conclusions

The objective of this study was to understand as rationally as possible the durability of GFRP rebars exposed to alkaline environments. In this study, GFRP rebars were aged in different aggressive conditions (direct immersion in an alkaline solution at pH 13.5 or indirect aging with an intermediate layer of concrete), and the main physical, mechanical and bond properties were monitored during aging. Complementary characterizations were also carried out by SEM, EDX, DSC, FTIR and mass/diameter monitoring, in order to relate the observed evolutions of macroscopic properties to possible microstructural changes or degradations. 

After the direct immersion of the GFRP rebars in the alkaline solution, a significant reduction in the longitudinal tensile strength was observed (of more than 40% after immersion at 60 °C for 240 days), which was correlated to the degradations of the glass fibers and the fiber/matrix interface in the outermost region/layer of the bars, as evidenced by SEM observations. A predictive model, based on the Arrhenius law, then made it possible to extrapolate the loss of tensile strength over a period of approximately 2.5 years at a reference temperature of 20 °C. However, this type of accelerated aging by direct immersion remains much more severe than exposition to the actual concrete environment where the mobility of hydroxyl ions is rather limited. SEM observations showed much less degradation on rebars exposed to indirect aging (embedded in concrete before immersion in the alkaline solution) compared to direct immersion. Subsequently, it could be interesting to investigate in a further study the evolution of the tensile properties of rebars subjected to indirect aging. This environment would thus allow a reasonable diffusion kinetics of alkalis from the solution to the rebar via the porosity of concrete.

Globally, whatever the considered aging environment, no significant degradation of the vinylester matrix of GFRP rebars was detected (no change in the glass transition temperature, nor evidence of hydrolytic degradation by FTIR). In addition, the monitoring of the mass/diameter of GFRP rebars did not reveal any notable variation during aging. These observations, together with EDX analyses, allow the conclusion that the absorption of the solution by the GFRP rebars remains very limited despite the great aggressiveness of the imposed accelerated aging conditions. Likewise, very little change in the interlaminar shear strength was observed after aging (an increase in ILSS was even highlighted for rebars subjected to direct immersion), indicating good chemical resistance of the vinylester matrix and the fiber/vinylester matrix interface in the core rebar.

Finally, several conclusions were drawn regarding the evolution of the bond properties of GFRP rebars in concrete during aging. The initial increase in bond strength observed in the first stage of exposure (from 0 to 120 days), which differs from trends usually described in the literature, has been assigned to the evolution of the concrete properties (post-curing, shrinkage or concrete swelling reactions) that produces additional confinement of the GFRP rebar. However, in a second stage (between 120 days and 240 days of exposure), the results of pull-out tests highlight a decreasing trend of the bond strength, suggesting that the degradation process at the concrete/rebar interface becomes predominant over the other beneficial effects. Further research is needed to validate this hypothesis, in particular durability studies conducted over longer exposure times. Nevertheless, the present results can be considered optimistic since the bond strengths of aged specimens (up to 240 days exposure) are above that of unaged reference specimens.

## Figures and Tables

**Table 1:** pH of alkaline solutions in the thermoregulated tanks (data from Ref. [34]).

Environment	pH (t = 0)	pH (t = 240 Days)
20-AK	13.6	13.0
40-AK	13.5	13.2
60-AK	13.6	13.1

**Table 2:** Distribution of the test specimens in the various accelerated aging environments.

Environment	Rebars for Tensile Properties	Rebars for ILSS	Rebars for Bond Properties	Rebars for Mass/ Diameter Monitoring	Number of Samples
20-AK	20-AK-D-120D				5
	20-AK-D-240D				5
		20-AK-D-120D			3
		20-AK-D-240D			3
		20-AK-I-120D			3
		20-AK-I-240D			3
			20-AK-I-120D		4
			20-AK-I-240D		4
				20-AK-D-MASS-S	3
				20-AK-D-MASS-NS	1
40-AK	40-AK-D-120D				5
	40-AK-D-240D				5
		40-AK-D-120D			3
		40-AK-D-240D			3
		40-AK-I-120D			3
		40-AK-I-240D			3
			40-AK-I-120D		4
			40-AK-I-240D		4
				40-AK-D-MASS-S	3
				40-AK-D-MASS-NS	1
60-AK	60-AK-D-120D				5
	60-AK-D-240D				5
		60-AK-D-120D			3
		60-AK-D-240D			3
		60-AK-I-120D			3
		60-AK-I-240D			3
			60-AK-I-120D		4
			60-AK-I-240D		4
				60-AK-D-MASS-S	3
				60-AK-D-MASS-NS	1
40-T		40-T-120D			3
		40-T-240D			3
			40-T-120D		4
			40-T-240D		4
60-T		60-T-120D			3
		60-T-240D			3
			60-T-120D		4
			60-T-240D		4
TOTAL					130

**Table 3:** Mass variations of GFRP rebars subjected to direct immersion in the alkaline solution.

Temperature	Sample	Mass Gain (%)			
		30 Days	60 Days	90 Days	120 Days
20 °C	20-AK-D-MASS-S-1	1.07	0.67	0.80	0.53
	20-AK-D-MASS-S-2	0.82	0.44	0.49	0.27
	20-AK-D-MASS-S-3	1.39	0.55	0.50	0.28
	20-AK-D-MASS-NS-1	0.25	0.31	0.00	0.63
40 °C	40-AK-D-MASS-S-1	1.39	0.80	0.64	0.55
	40-AK-D-MASS-S-2	1.42	0.77	0.60	0.57
	40-AK-D-MASS-S-3	1.32	0.47	0.45	0.53
	40-AK-D-MASS-NS-1	−0.31	0.56	0.63	0.44
60 °C	60-AK-D-MASS-S-1	1.66	0.30	0.33	0.28
	60-AK-D-MASS-S-2	1.14	0.37	0.23	0.28
	60-AK-D-MASS-S-3	1.13	0.45	0.39	0.28
	60-AK-D-MASS-NS-1	−0.46	−0.52	−0.62	−0.37

**Table 4:** Diameter variations of GFRP rebars subjected to direct immersion in the alkaline solution.

Temperature	Type of Rebar	Measure No.	Diameter Variation (%)			
			30 Days	60 Days	90 Days	120 Days
20 °C	Sand-coated	1	−2.84	−0.43	−2.84	−2.84
		2	−0.72	1.73	−2.88	−3.45
		3	−1.43	2.50	−3.93	−2.29
		Mean	−1.7 ± 0.9	1.3 ± 1.3	−3.2 ± 0.6	−2.9 ± 0.5
	Non-coated	1	1.79	−0.81	−0.81	−0.57
		2	1.63	−0.24	−0.65	−1.22
		3	2.54	0.66	0.41	0.25
		Mean	2.0 ± 0.4	−0.1 ± 0.6	−0.4 ± 0.6	−0.5 ± 0.6
40 °C	Sand-coated	1	−1.42	−0.64	−4.61	−5.18
		2	−2.14	0.00	−5.71	−4.29
		3	−6.12	−4.22	−7.21	−3.40
		Mean	−3.2 ± 2.1	−1.6 ± 1.9	−5.9 ± 1.1	−4.3 ± 0.8
	Non-coated	1	0.81	−1.61	−2.02	−1.69
		2	1.63	−1.22	−0.41	−0.81
		3	1.87	−1.06	−1.06	−0.81
		Mean	1.4 ± 0.5	−1.3 ± 0.3	−1.2 ± 0.7	−1.1 ± 0.5
60 °C	Sand-coated	1	−1.45	1.38	−2.90	−1.09
		2	−3.62	−0.51	−3.99	−2.17
		3	−0.73	0.36	−2.19	−3.28
		Mean	−1.9 ± 1.3	0.4 ± 0.8	−3.2 ± 0.8	−2.2 ± 0.9
	Non-coated	1	2.46	−0.33	0.82	0.16
		2	1.30	−1.14	1.63	−0.57
		3	−0.87	−3.57	−1.59	−1.90
		Mean	1.0 ± 0.4	−1.7 ± 1.4	0.3 ± 1.4	−0.8 ± 0.9

**Table 5:** Values of the glass transition temperature determined by DSC analyses on reference GFRP rebars (REF) and on rebars exposed for 240 days to direct immersion in the alkaline solution at various temperatures. Values obtained at first and second runs are reported (*T_g_* (1) and *T_g_* (2)).

Localization	*T_g_*	Sample No.	Aging Condition			
			REF	20-AK-D	40-AK-D	60-AK-D
Close to the surface	*T_g_* (1) (°C)	1	114.7	116.9	115.1	115.3
		2	118.7	115.8	115.8	114.9
		3	116.8	117.0	114.5	114.1
		Mean	117 ± 2	116.6 ± 0.6	115.1 ± 0.5	114.8 ± 0.5
	*T_g_* (2) (°C)	1	128.7	128.6	126.3	129.1
		2	128.1	128.3	126.0	130.2
		3	128.2	128.4	126.6	129.4
		Mean	128.3 ± 0.2	128.4 ± 0.1	126.3 ± 0.2	129.6 ± 0.4
Core of the rebar	*T_g_* (1) (°C)	1	115.9	-	-	114.5
		2	117.5	-	-	115.2
		3	116.0	-	-	114.9
		Mean	116.5 ± 0.7	-	-	114.9 ± 0.3
	*T_g_* (2) (°C)	1	127.8	-	-	129.7
		2	125.7	-	-	129.0
		3	129.3	-	-	129.8
		Mean	128 ± 2	-	-	129.5 ± 0.4

**Table 6:** Results of tensile tests performed on GFRP rebars exposed to direct immersion in the alkaline solution. Results obtained for unaged reference rebars are also reported (REF).

Aging Duration	Sample No.	Elastic Modulus (GPa)				Tensile Strength (MPa)			
		REF	20-AK-D	40-AK-D	60-AK-D	REF	20-AK-D	40-AK-D	60-AK-D
0 days	1	51.3				1271			
	2	50.2				1272			
	3	50.5				1192			
	4	52.9				1232			
	5	51.3				1233			
	Mean	51 ± 1				1240 ± 29			
120 days	1		-	50.3	50.8		1082	962	812
	2		51.6	48.5	51.1		1158	968	792
	3		52.0	50.2	51.5		1183	965	827
	4		51.3	52.3	51.5		1157	981	829
	5		51.6	52.0	53.7		1176	983	811
	Mean		51.6 ± 0.2	51 ± 2	52 ± 1	-	1151 ± 36	972 ± 9	814 ± 13
240 days	1		51.2	50.7	50.2		1065	848	761
	2		53.3	51.3	48.0		1034	925	642
	3		51.9	44.8	44.7		1087	653	658
	4		51.5	50.9	51.7		1083	922	890
	5		51.7	50.8	43.2		1107	877	625
	Mean		51.9 ± 0.7	49.7 ± −2.5	48 ± 4	-	1075 ± 24	845 ± 100	715 ± 99

**Table 7:** Time shift factors determined from the experimental results at 20 °C, 40 °C and 60 °C.

**Reference Temperature *T*_0_**	***E_a_*/*R***
20 °C	3500 K
**Temperature *T*_1_**	**Time shift factor *TSF_T_*_1*/T*0_**
40 °C	2.14
60 °C	4.19

**Table 8:** Results of short-beam tests performed on GFRP rebars exposed to direct (AK-D) and indirect (AK-I) immersion in the alkaline solution, and to pure thermal aging as well (T). Results are also provided for unaged reference rebars (REF).

Time	Sample No.	Interlaminar Shear Strength (MPa)								
		REF	20-AK-D	20-AK-I	40-AK-D	40-AK-I	40-T	60-AK-D	60-AK-I	60-T
0 days	1	44.43								
	2	45.53								
	3	46.90								
	Mean	46 ± 1								
120 days	1		48.85	46.01	46.80	47.22	43.95	52.64	46.11	39.74
	2		48.90	41.85	47.06	45.27	40.22	54.01	44.90	45.01
	3		44.37	42.27	48.27	40.58	38.74	53.06	42.43	44.32
	Mean		47 ± 3	43 ± 2	47.4 ± 0.7	44 ± 3	41 ± 3	53.2 ± 0.6	44 ± 2	43 ± 3
240 days	1		49.32	42.37	49.11	40.16	40.16	45.16	47.95	39.79
	2		49.80	46.74	54.27	46.01	43.58	40.00	50.01	45.90
	3		50.59	41.01	53.32	38.95	40.64	44.27	41.79	44.06
	Mean		49.9 ± 0.6	43 ± 3	52 ± 3	42 ± 4	41 ± 2	43 ± 3	47 ± 4	43 ± 3

**Table 9:** Results of compressive tests on reference and aged concrete cylinders.

Sample No.	Compressive Strength of Concrete (MPa)					
	REF	20-AK	40-AK	60-AK	40-T	60-T
1	29.86	37.75	37.30	26.95	34.95	24.90
2	29.35	37.75	36.30	25.70	36.70	34.25
3	28.95	37.30	36.75	26.60	37.75	33.45
Mean	29.4 ± 0.4	37.6 ± 0.3	36.8 ± 0.5	26.4 ± 0.6	36 ± 2	31 ± 5

**Table 10:** Results of pull-out tests performed on reference and aged specimens.

Time	Sample No.	Bond Strength (MPa)					
		REF	20-AK-I	40-AK-I	60-AK-I	40-T	60-T
0 days	1	6.23					
	2	6.61					
	3	5.81					
	4	6.15					
	Mean	6.2 ± 0.3					
120 days	1		6.29	7.66	7.47	7.81	9.92
	2		7.86	8.64	7.24	6.22	9.07
	3		7.82	7.07	5.89	10.83	9.37
	4		7.06	9.87	6.91	7.54	9.00
	Mean		7.3 ± 0.7	8 ± 2	6.9 ± 0.6	8 ± 2	9.3 ± 0.4
240 days	1		8.51	7.00	6.83	5.73	7.79
	2		6.13	6.32	8.13	7.94	7.78
	3		7.33	8.91	7.27	7.61	-
	4		6.91	6.76	7.71	7.07	7.33
	Mean		7.2 ± 0.9	7 ± 1	7.5 ± 0.5	7.1 ± 0.9	7.6 ± 0.3

## Data Availability

Not applicable.
